# Ankyloblepharon-ectodermal defects-cleft lip/palate syndrome in monozygotic twins with a novel tumor protein p63 gene pathogenic variant

**DOI:** 10.1016/j.jdcr.2025.04.003

**Published:** 2025-04-14

**Authors:** Ibeth Caceres, Jennifer Good, Clare Asher, Elizabeth A. Sellars, Megan S. Evans

**Affiliations:** a Baylor College of Medicine, Houston, Texas; b Department of Dermatology, University of Arkansas for Medical Sciences, Little Rock, Arkansas; c Section of Neonatology, Department of Pediatrics, University of Arkansas for Medical Sciences, Little Rock, Arkansas; d Arkansas Children’s Hospital, Little Rock, Arkansas; e Section of Genetics and Metabolism, Department of Pediatrics, University of Arkansas for Medical Sciences, Little Rock, Arkansas

**Keywords:** AEC syndrome, ectodermal dysplasia, genodermatoses, monozygotic twins, pediatric dermatology

## Introduction

Ankyloblepharon-ectodermal defects-cleft lip/palate (AEC) syndrome is a rare genodermatosis within the tumor protein p63 gene (*TP63*)–related ectodermal dysplasia spectrum. *TP63* encodes the transcription factor p63, which is critical for the differentiation, proliferation, and maintenance of ectodermal structures, including the epidermis, hair, nails, and teeth.^1^ AEC syndrome is characterized by a range of clinical findings, including ankyloblepharon, neonatal erythroderma, erosive scalp dermatitis, limb malformations, hypohidrosis, hypodontia, nail abnormalities, sparse hair, and cleft lip/palate.^1^ *TP63*-related disorders are believed to arise from dominant-negative or gain-of-function pathogenic mechanisms affecting the p63 protein.^2^ However, recent studies challenge this view by demonstrating that loss-of-function variants in *TP63* can also contribute to the broad clinical phenotype observed within this spectrum of disorders.^2^ In this report, we describe a novel pathogenic variant in monozygotic twins with AEC syndrome with phenotypic discordance.

## Case report

Female monozygotic twins were delivered via cesarean section at 37 weeks of gestation without complications. Twin A weighed 2.17 kg and twin B weighed 2.45 kg. Family history was negative for congenital anomalies including cutaneous diseases. The pregnancy was complicated by hydronephrosis in both twins.

**Fig 1:**
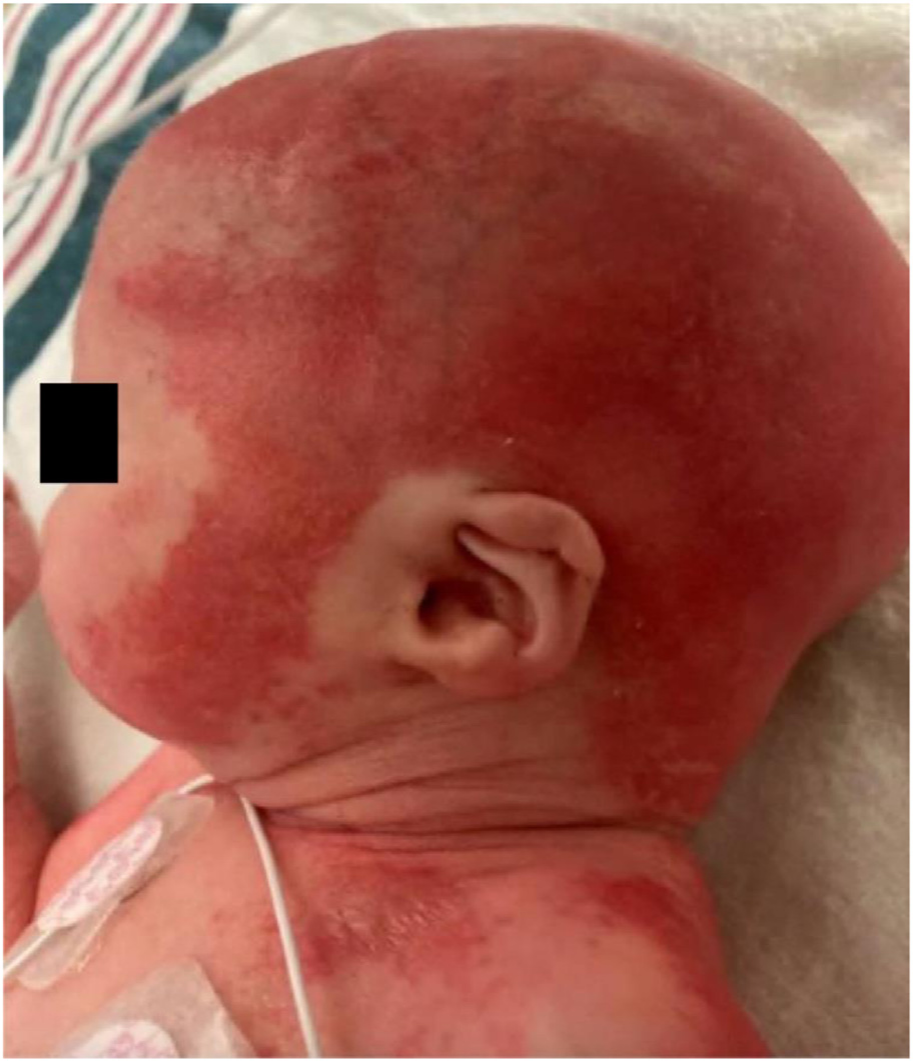
AEC syndrome. Scalp erosions and complete absence of hair in twin B. *AEC*, Ankyloblepharon-ectodermal defects-cleft lip/palate.

**Fig 2:**
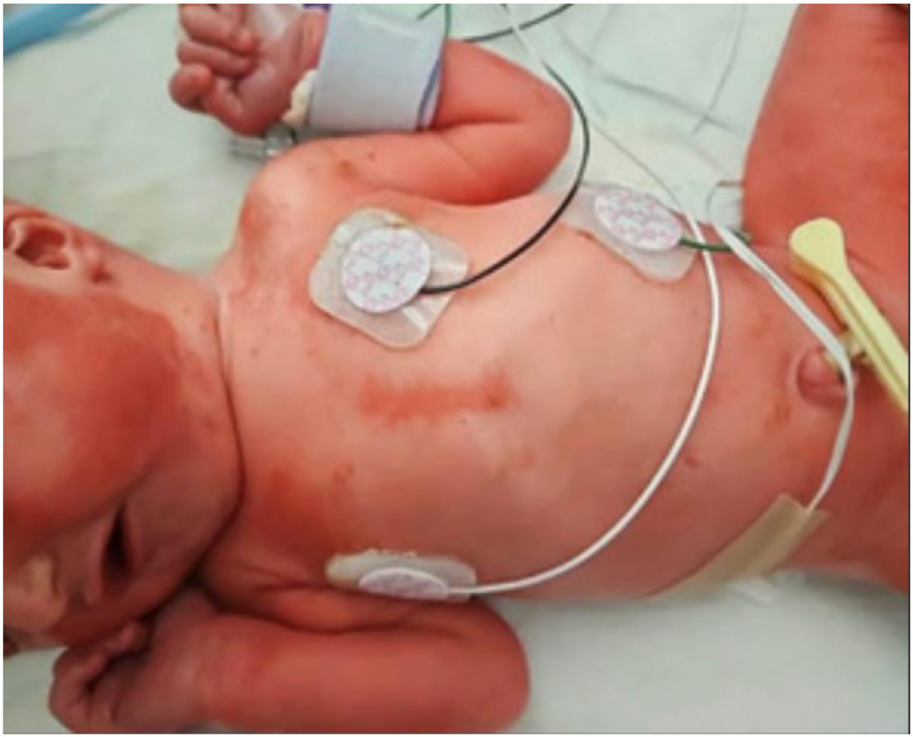
AEC syndrome. Erosions over the upper and lower extremities in twin A. *AEC*, Ankyloblepharon-ectodermal defects-cleft lip/palate.

Physical examination of both twins revealed a complete absence of scalp hair, eyebrows, and eyelashes, although nails appeared normal. Diffuse erythema, scalp erosions, and extensive desquamation affecting the chest, back, and lower extremities with sparing of the hands and feet were noted (Figs 1 and 2). Additional findings included Veau Class II cleft palate and a combination of micrognathia, glossoptosis, and upper airway obstruction, consistent with Pierre Robin sequence. Twin A exhibited mild right-sided conductive hearing loss.

After delivery, broad-spectrum empiric antibiotics and acyclovir were initiated pending infectious and genetic workup. Genetic analysis identified a *de novo* heterozygous, pathogenic variant in exon 11 of *TP63* (c.1198_1208delACCATTCCTGAinsTGT; p.Thr400 fs). This frameshift variant introduces a premature termination codon in exon 11 of *TP63.*

Management was supportive and involved continuous monitoring for electrolyte imbalances, temperature derangement, and infection. Twin A experienced significant feeding difficulties secondary to retrognathia, necessitating surgical intervention. Skin-specific supportive care included the use of a humidifier with gradual weaning as tolerated, gentle skin care with synthetic detergent, and liberal application of white petrolatum.

## Discussion

AEC syndrome is an autosomal dominant disorder associated with pathogenic variants in the sterile-alpha-motif and transactivation inhibitory domains of p63, primarily involving missense and frameshift variants in exons 13 and 14.^1^^,^^3^^,^^4^ However, nonsense mutations leading to truncated forms of p63 have been documented. Some of these variant proteins, which escape nonsense-mediated mRNA decay, retain functional domains, refuting the haploinsufficiency model and supporting the idea that *TP63* variants primarily function through dominant-negative or gain-of-function pathogenic mechanisms.^2^^,^^5^

Genetic testing revealed a novel pathogenic variant in *TP63* that has not been previously reported in the literature or documented in open access genetic variant databases, including Leiden Open Variation Database, ClinVar, and genome aggregation database. This case is particularly significant as it not only presents a rare occurrence of AEC syndrome in monozygotic twins but also contributes to the genetic understanding of the disorder. The identified variant is located outside the commonly reported exons 13 and 14, affecting the second transactivation domain, TA2, rather than the frequently implicated sterile-alpha-motif and transactivation inhibitory domains.^5^ This finding highlights the genetic heterogeneity of AEC syndrome.

Only 1 other case of AEC syndrome in monozygotic twins has been reported in the literature.^6^ The monozygotic female twins described here possess a novel *de novo* heterozygous, pathogenic variant in the *TP63* gene and are concordant for cutaneous erosions, absence of hair, cleft palate, and hydronephrosis. However, they exhibited discordance in the severity of Pierre Robin sequence and hearing loss. As observed in this case, monozygotic twins with genetic syndromes can exhibit phenotypic discordance despite sharing identical genetic material. This variability may be attributed to epigenetic differences, environmental influences, stochastic factors, and postzygotic mutations leading to genetic mosaicism.^7^ Phenotypic heterogeneity in *TP63-*related disorders among monozygotic twins has been previously documented.^6^^,^^8^ This case advances the current understanding of AEC syndrome by underscoring and expanding the clinical and genetic spectrum of the disorder.

Dermatology plays a pivotal role in the identification and evaluation of ectodermal dysplasias, as these disorders may manifest with prominent cutaneous findings at birth. Dermatologists are often the first specialists to assess these features, facilitating early diagnosis and timely interdisciplinary collaboration among geneticists, otolaryngologists, orofacial specialists, and neonatologists. Dermatologic care, including optimization of the skin barrier function and wound care, is essential to improving clinical outcomes and enhancing patient quality of life. Beyond the neonatal period, dermatologists contribute to ongoing care by addressing sequelae of ectodermal dysplasias, such as recurrent infections, atopic dermatitis, and alopecia, and providing psychosocial support to mitigate their impact on families.^9^ This case highlights the importance of dermatologic expertise in diagnosing and managing ectodermal dysplasias and emphasizes the need for a multidisciplinary approach.

## Conflicts of interest

None disclosed.
